# Eye-tracking metrics to compare visual attention in prosthodontic preclinical evaluations

**DOI:** 10.1186/s12903-025-06708-6

**Published:** 2025-08-21

**Authors:** Frédéric Silvestri, Abhishek Kumar, Maria Christidis, Anastasios Grigoriadis

**Affiliations:** 1 Department of Prosthodontics, School of Dental Medicine, ADES, CNRS, EFS, Aix-Marseille University, Marseille, France; 2 Division of Oral Rehabilitation, Department of Dental Medicine, Karolinska Institutet, Box 4064, 141 04 Huddinge, Alfred Nobels Allé 8, Huddinge, Sweden; 3 Academic Center for Geriatric Dentistry, Stockholm, Sweden; 4 Department of Neurobiology, Care Sciences and Society, Karolinska Institutet, Huddinge, Sweden; 5 Department of Nursing, Sophiahemmet University, Stockholm, Sweden

**Keywords:** Tooth preparation, Gaze behavior, Prosthodontics education, Cognitive load, Visual processing skills

## Abstract

**Aim:**

The study aimed to evaluate gaze behavior during tooth preparation assessments by analyzing and comparing eye-tracking metrics between novice and expert groups.

**Methods:**

Thirty-five participants, divided into novices (*n* = 18, mean age = 22.9 ± 1.5 years) and experts (*n* = 17, mean age = 44.3 ± 13.1 years), were recruited for this observational study. The novice group consisted of third-year dental students, while the expert group comprised licensed dentists with an average of 18.9 ± 12.7 years of clinical experience. Eye-tracking metrics, including total duration of fixation (TDF), number of fixations (NF), time to first fixation (TFF), and pupil size, were measured across different areas of interest (AOIs). The data was analyzed with a two-way repeated measures analysis of variance (ANOVA) model.

**Results:**

Both novices and experts focused mainly on the “buccal wall” and “margin” (finishing line) AOIs during tooth preparation evaluation. The novices showed significantly longer TDF (*P* = 0.034), more NF (*P* = 0.047), and longer TFF (*P* = 0.021) compared to experts. However, there were no significant differences in pupil diameter between groups or AOIs, indicating similar cognitive load despite differences in visual behavior.

**Conclusion:**

Overall, the novices tend to have longer fixation durations, more frequent fixations, and a delayed time to first fixation compared to experts during tooth preparation assessments. The study also concludes that both novices and experts primarily focus on the buccal wall and finishing line. These differences indicate that visual processing varies between the two groups, with novices demonstrating less efficient visual processing skills. In general, the findings highlight how experience influences gaze behavior in the assessment of tooth preparation.

**Clinical significance:**

These findings can refine pre-clinical prosthodontic education by fostering expert-like visual processing skills, enabling students to better understand and perform prosthodontic tasks. This targeted approach enhances their training and prepares them more effectively for clinical practice.

**Supplementary Information:**

The online version contains supplementary material available at 10.1186/s12903-025-06708-6.

## Background

Over the past few decades, several authors have carried out research into gaze behavior to gain a better understanding of eye movements and the relationship between the brain and the eyes [1–3]. Eye tracking is a sensor technology which records eye movements, based on corneal reflection and stereo geometry and can be found in both table and head mounted configuration. Over the last decade, and thanks to improvements in technology, eye tracking devices have been increasingly used in many different disciplines. These studies mainly investigated gaze behavior in different situations to better understand and interpret the cognitive workload involved in different types of tasks [4, 5]. 

Gaze behavior can mainly be characterized by two kinds of eye movements: fixations and saccades. A fixation is a period during which the eyes are fixed on a visual target, perception is stable, and the eyes are taking in detailed visual information by holding the foveal area in the same place. Saccades are rapid, ballistic movements of the eye from one fixation to the next [6, 7]. Several studies have shown that different metrics of gaze behavior could be used in a better understanding of cognitive workload in different tasks [7–9]. Researchers need to define specific areas of interest (AOIs) in advance, so the software can generate AOI-based metrics, such as how long or how often individuals focus on a particular area.

Eye tracking is a versatile tool that has also been used in different branches of medicine. In dentistry, eye trackers have been used to analyze in real time gaze behavior, mostly about visual perception in various situations such as perception of epitheses, esthetics, radiographs etc [10–13].,. In fixed prosthodontics, single crown tooth-preparation is a fundamental skill. The quality of tooth preparations is typically assessed using several key parameters, including the degree of preparation taper, the precision of the finish line, the depth of occlusal and axial reduction, the texture of the surface, and the form of line angles [14, 15]. Although there is no universal agreement on the exact standards for each of these parameters, they are widely taught by educators as critical criteria for evaluating student performance and proficiency in tooth preparation. The eye-tracking device can be a valuable tool for comparing the gaze behavior of students (novices) and teachers (experts) when assessing tooth preparation. By analyzing both conscious and unconscious visual attention, the device provides insights into how these two groups differ in their evaluation processes [7, 9, 16]. For example, when evaluating a single crown tooth preparation, the eye-tracking device can generate a vast array of metrics, such as total duration of fixation, time to first fixation, and the number of fixations for each predefined AOI. Focusing on a few key metrics can provide more meaningful insights into the objective assessment of tooth preparation [7]. 

Experience often leads to more refined and efficient cognitive processing strategies. Experienced individuals likely have a more developed schema or mental model for tasks within their domain, enabling them to quickly identify relevant information and make decisions with less cognitive effort [17, 18]. In contrast, novices may rely on more effortful and less efficient strategies, leading to differences in eye-tracking metrics that reflect greater uncertainty and exploration. Previous studies have suggested that pupil diameter fluctuations are related to cortical arousal and cognitive workload [19]. Thus, investigating various eye-tracking metrics is essential for understanding gaze patterns more comprehensively.

We have recently shown that gaze behavior, measured through eye tracking, is an indicator of how individuals process information in dental education [20]. We have also preliminarily shown that the undergraduate dental students (novices) exhibited significantly longer duration of fixation compared to the expert group, and the duration of fixation was dependent on the views, AOIs, and the quality of tooth preparation [20]. Building on these findings, our current study further aims to evaluate additional eye-tracking metrics, including the number of fixations, time to first fixation, and pupil size. We will compare these metrics between novice (undergraduate students) and expert (educators/dentists) groups across more AOIs to gain a deeper understanding of their gaze behavior. It was hypothesized that the novices would show a longer duration of fixation, a greater number of fixations, and a longer time to first fixation compared with the experts.

## Materials and methods

Participants for both groups were randomly selected and recruited from the Department of Dental Medicine, Karolinska Institutet, Sweden. Informed written consent was obtained from each participant in accordance with the Declaration of Helsinki. Participants were made aware of their right to withdraw from the study at any time, without the need to provide a reason. The study was reviewed and approved by the Ethics Review Authority in Stockholm (Dnr 2023-04136-01)

### Study participants

A total of thirty-five participants, divided into two groups, volunteered to participate in the current observational study. The groups comprised a novice group (*n* = 18, mean age = 22.9 ± 1.5 years) and an expert group (*n* = 17, mean age = 44.3 ± 13.1 years). A priori power analysis was conducted using G*Power to determine the required sample size for an ANOVA with repeated measures and within-between interaction. Assuming a medium effect size (f = 0.3), an alpha level of 0.05, and a desired statistical power of 0.90, the analysis indicated that a total of 32 participants would be needed to achieve an actual power of approximately 0.91. The novice group consisted of third-year undergraduate students, while the expert group included licensed dentists who were also faculty members teaching preclinical prosthodontics. The experts had a mean experience of 18.9 ± 12.7 years in active clinical practice at the time of the study.

### Study setting

**Fig. 1:**
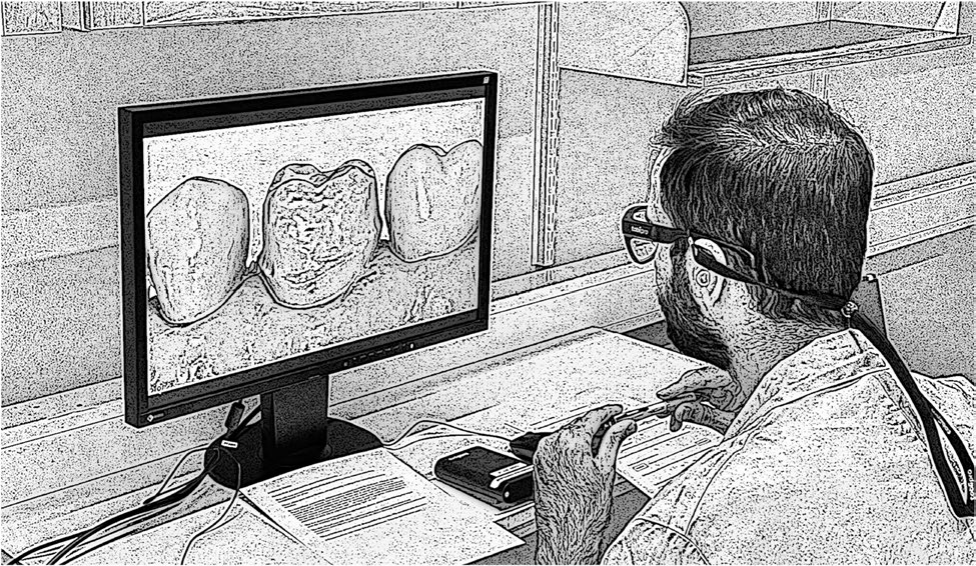
Experiment setup

The study was conducted in accordance with the Reporting Standards for Eye-tracking Data in Empirical Studies (RESIDE) recommendations by Cho et al. that are developed to standardize the environment, to limit bias, and improve the reporting quality of eye-tracking studies [21]. The experiment was conducted in a quiet room, isolated from external noise, and illuminated with regular artificial light (3000 K). The tooth preparation pictures were displayed on a screen (FlexScan^®^ EV2416W, 24.1 inches, 1920 × 1080 pixels, 50–60 Hz; Ezio Corporation, Japan) placed on a desk in front of a monochrome blank wall. The participants were seated on an adjustable chair and instructed to position themselves so that they were looking horizontally at the screen. They wore a binocular eye-tracking device (Tobii Pro Glasses 3^®^, Danderyd, Stockholm, Sweden) throughout the experiment. Please see Fig. 1 for the illustration of the experimental setup. The examiner assisted the participants in carefully fitting the wearable eye tracker, which was worn like a pair of spectacles, following the manufacturer’s recommendation to maintain a distance of 0.75 to 1 m from the screen. The eye-tracking system included a calibration procedure and had a gaze position accuracy of 0.6°. To ensure maximum silence, participants were also asked to wear earplugs during the experiment. The video recordings were captured using dedicated software (Glasses 3 controller^®^, Danderyd, Stockholm, Sweden; Tobii AB). Throughout the experiment, the examiner remained in the room to monitor the process, ensuring that the experiment proceeded smoothly while maintaining a discreet presence to avoid influencing the participants’ behavior.

### Specimen preparation

Twenty acrylic right first maxillary molar teeth (Frasaco^®^, Franz Sachs GMBH & Co, Germany) were prepared for full-zirconia crowns by one of the investigators (FS). Digital scanning was performed using an intraoral scanner (Cerec^®^ Omnicam, Dentsply Sirona, Charlotte, United States). From the scan file of each tooth preparation, three high-resolution images (1920 × 1080 pixels) were selected, capturing the buccal, lingual, and occlusal views. These images were carefully chosen to represent the different aspects of each tooth preparation. Thus, in total, sixty photographs were prepared, representing twenty different tooth preparations in three distinct views. However, only twenty pictures from the buccal view were taken for further in-depth analysis of the eye tracking metrics, which included the total duration of fixation, the number of fixations, time to first fixation, and pupil size

### Experimental protocol

All participants were briefed about the main aim of the study and were given written and verbal instructions on how the study would be conducted. The experimental session commenced with a practice trial that was excluded from the analysis. The practice session allowed participants to familiarize themselves with the task and ask clarifying questions (if any). The participants then observed all twenty pictures one by one. The display duration was 15 s for each picture, but the participants could move on to the next picture without waiting for the allotted 15 s, with no possibility of returning. To minimize fatigue during the assessment, participants were also given a one-minute break after every five tooth preparations were displayed. This structured approach ensured that participants were well-prepared and maintained their focus throughout the experimental session.

### Data analysis

**Fig. 2:**
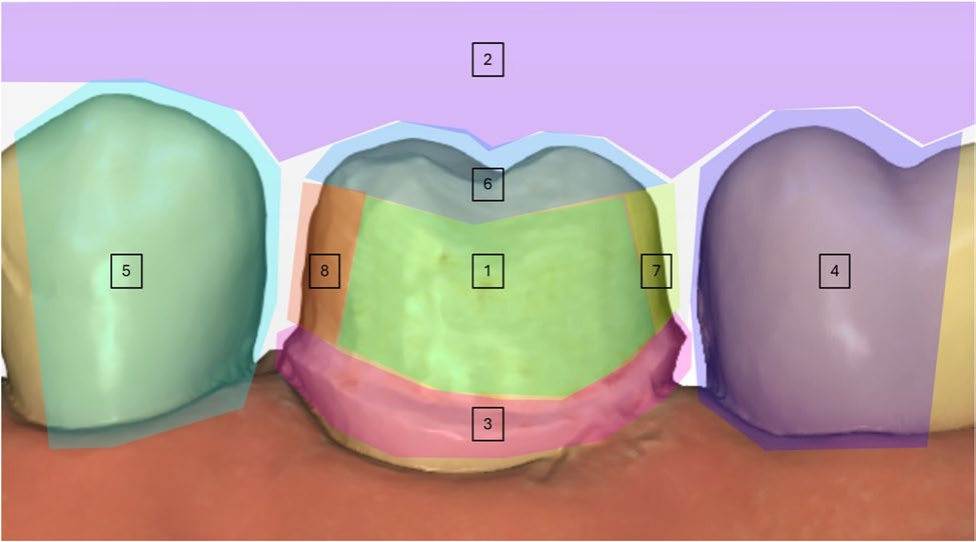
Example of different areas of interest drawn on the buccal view (1 = buccal wall, 2 = height, 3 = margin, 4 = distal adjacent, 5 = mesial adjacent, 6 = occlusal, 7 = distal taper, 8 = mesial taper)

All the collected video files were analyzed using a dedicated software (Tobii Pro Lab^®^, v 1.217, [Computer software]; Danderyd, Stockholm, Sweden: Tobii AB). The buccal view was divided into 8 “areas of interest’’ (AOI), which included the margin, mesial taper, distal taper, occlusal shape, buccal or lingual wall, mesial adjacent tooth, distal adjacent tooth, and height which were outlined in the software (Fig. 2). The fixation threshold was set at 200 ms. For each participant and each AOI, the total duration of fixations (TDF), time to first fixation (TFF), number of fixations (NF), and average pupil diameter were recorded.

### Statistical analysis

The data was analyzed with version 27, SPSS (Statistical Package for the Social Sciences, IBM, Inc.). The interval duration (15 s.) corresponded to the display time of the buccal view (see Experimental Protocol). The percentage of time each participant spent looking at an AOI was calculated by dividing the TDF by the interval duration. The mean TDF across the twenty photographs displayed for each AOI was then calculated and used for analysis. The data was assessed for normality using the Shapiro-Wilk test, as well as by examining histograms and Q-Q plots. To prevent the loss of zero values, a small constant of 0.1 was added to the entire dataset, except for the pupil diameter measurements. The TDF was defined as the cumulative time spent on a specific area, while TFF was the time elapsed from the start of the display of an image to the first fixation of gaze. NF was defined as the total number of fixations on a specific area. Following this adjustment, the data was logarithmically transformed to stabilize variance and normalize the distribution, improving the suitability for further analysis. The data related to the outcome variables (total duration of fixation, number of fixations, time to first fixation, and pupil diameter) were analyzed using a two-way analysis of variance (ANOVA) model with repeated measures. This analysis was designed to evaluate the differences in outcome parameters based on two factors: groups (two levels: novices and experts), and AOI (8 levels: buccal wall, height, margin, mesial adjacent tooth, distal adjacent tooth, occlusal, mesial taper, distal taper). Following the ANOVA, post hoc comparisons of significant main effects were conducted using the Tukey Honestly Significant Difference (HSD) test for multiple pairwise comparisons between the means. In this analysis, a *P*-value of less than 0.05 was used as the criterion for statistical significance, ensuring that the findings are robust and not due to random variation.

## Results

**Fig. 3:**
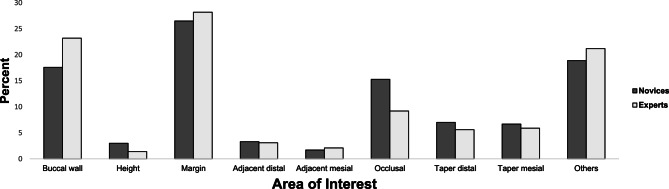
Percentage distribution of total duration of fixation according to the area of interest

All the participants in the group were able to complete the entire experimental session without any discomfort in a reliable manner. In general, during the allocated 15 s to evaluate the tooth preparation, both novices and experts primarily focused on the “buccal wall” and “margin” AOIs. The “Buccal wall” accounted for 17.6% of the total duration of fixations (TDF) for novices and 23.2% for experts. The “Margin” represented 26.5% of the TDF for novices and 28.2% for experts. Additionally, the total duration of fixations outside the predefined AOIs was 18.9% for novices and 21.2% for experts. In contrast, the total duration of fixations within the overall AOIs of tooth preparation was 81.1% for novices and 78.8% for experts (Fig. 3).

### Total duration of fixation (TDF)

**Fig. 4:**
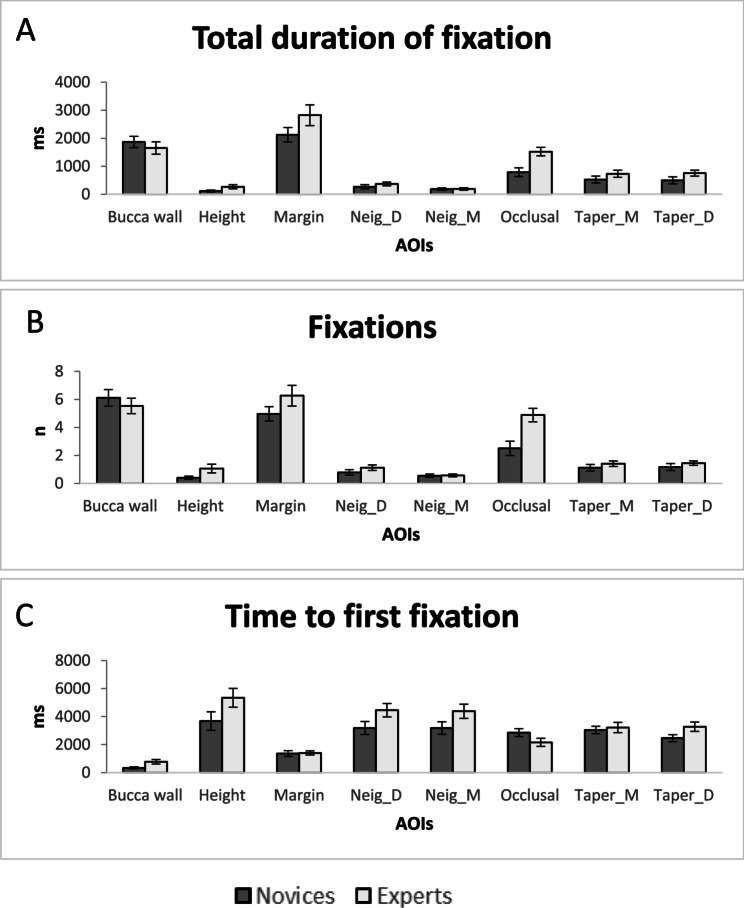
Mean and standard error of the mean (SEM) of the (**A**) total duration of fixation, (**B**) the number of fixations, and (**C**) time to first fixation for the novice and expert groups

The results of ANOVA analysis showed significant main effects of groups (novices/experts) (*P* = 0.034) and AOIs (buccal wall, height, margin, mesial adjacent tooth, distal adjacent tooth, occlusal, mesial taper, distal taper) (*P* < 0.001) (Fig. 4A). Post hoc analysis of the main effects of groups showed significantly longer TDF in the novices compared to the experts. Post hoc analysis of the main effects of the AOI showed significantly longer TDF in “buccal wall” and “margin” compared to other AOIs except “occlusal” (*P* < 0.001). Post hoc analysis of the main effects of the AOIs also showed significantly shorter TDF in “height” compared to all the other AOIs (*P* < 0.003).

The results of the ANOVA also showed significant interactions between AOIs and group (*P* = 0.001). Post hoc analysis of the AOIs and group showed significantly higher TDF in the novices compared to the experts while observing the height (*P* < 0.001)

### The number of fixations (NF)

Similar to the TDF, the results of ANOVA analysis on NF showed significant main effects of groups (*P* = 0.047) and AOIs (*P* < 0.001) with significant interaction between the groups and AOIs (*P* = 0.006). Post hoc analysis of the main effects of groups showed significantly higher NF in the novices compared to the experts (*P* < 0.001). Post hoc analysis of the main effects of the AOIs showed significantly higher NF in “buccal wall” and “margin” (*P* < 0.002) compared to all the other AOIs. Post hoc analysis of the main effects of the AOIs also showed significantly lower NF in “height,” compared to other AOIs (*P* < 0.002) except “mesial adjacent tooth” (*P* = 0.993) (Fig. 4B).

### Time to first fixation (TFF)

The results of ANOVA analysis showed significant main effects of groups (*P* = 0.021) and AOIs (*P* < 0.001) but no significant interactions between the groups and AOI (*P* = 0.083). Post hoc analysis of the main effects of groups showed significantly longer TFF in the novices compared to the experts. Post hoc analysis of the main effects of AOIs showed a significantly shorter time to first fixation in the “buccal wall” (*P* < 0.001) compared to other AOIs, and significantly shorter TFF in “margin” compared to “mesial adjacent tooth” (*P* = 0.040) and “distal adjacent tooth” (*P* = 0.021) (Fig. 4C).

### Average pupil diameter

The results of the ANOVA analysis showed no significant effect of either the groups (*P* = 0.081) or the AOI (*P* = 0.424) or any significant interaction between the groups and AOI (*P* = 0.596) on the average pupil diameter.

## Discussion

Eye-tracking devices have been used in various fields to better understand how visual information is collected and analyzed by humans. It has also been used in various medical specialties to assess the gaze behavior of students or inexperienced practitioners to improve medical education [10–13]. In the current study, eye tracking was used to investigate the differences in gaze behavior between novices and experts while evaluating specific areas of interest in a single crown tooth preparation. In accordance with the hypotheses, the results showed significant differences in gaze behavior between novices, consisting of a group of undergraduate dental students, and experts, consisting of licensed dentists, while assessing single crown tooth preparation. Specifically, the results of the current study showed that the novices had a significantly longer total duration of fixations (TDF), more fixations (NF), and a longer time to first fixation (TFF) compared to experts. Both groups spent the most time on the “buccal wall” and the “margin” AOIs. However, there were neither significant differences in the pupil diameter between the groups nor significant changes in the pupil diameter while observing different AOIs. These results indicate that students (novices) and licensed dentists (experts) differ significantly in how they visually engage with specific AOIs during tooth preparation assessment. Novices tend to spend more time fixating on certain areas, have more fixations, and take longer to focus on critical regions compared to experts. However, there were no significant differences in pupil diameter between the groups or across different AOIs, suggesting similar cognitive load despite differences in visual engagement.

The images of the tooth preparation used in the study depicted various characteristics, including total occlusal convergence, finish line, and occlusal reduction, which were monitored and recorded using specialized software. Following the recommendations of the previous studies, the images were carefully selected to avoid any “attention-grabbing” elements that could affect fixation times [7]. The entire experimental procedure was conducted in accordance with the RESIDE recommendations, which took into account several parameters of the setup, such as technical aspects, participants, conditions, etc [21]. Standardizing the surrounding conditions can help reduce biases introduced by the experimental setup, and are positive attribute of the current study [7, 22]. Previous observations indicated that the duration spent assessing the buccal view was notably longer and appeared crucial for evaluating tooth preparation [20]. Consequently, we decided to conduct an in-depth analysis of the buccal view, focusing on various eye-tracking metrics and more AOIs. This approach allowed us to better understand visual attention and behaviors during the assessment process, providing more comprehensive insights into the evaluation criteria for tooth preparation.

Tooth preparation for a single crown is a fundamental skill in prosthodontic training, essential for students to master before graduation. Several studies have identified and examined the key aspects involved in both performing and evaluating tooth preparations. These studies highlight the critical techniques and considerations that ensure successful outcomes in crown placement, emphasizing their importance in dental education and practice. In the current study, the AOIs have been chosen according to the different zones that are of importance when assessing a single crown tooth preparation [15, 23, 24]. Accordingly, five AOIs (margin, mesial taper, distal taper, buccal wall, and occlusal) were drawn to cover the whole tooth preparation. Three other AOIs were drawn to analyze the participants’ gaze behavior on the adjacent teeth (mesial adjacent tooth, distal adjacent tooth) and occlusal reduction (height). The subject-based reports revealed that both novice and expert groups focused most on the “buccal wall” and “margin” as the primary AOIs. In this study, the buccal wall stood out as a larger and more prominent area, which naturally drew the participants’ attention at first. Consequently, their gaze was fixated on the buccal wall, making it a key focal point in the observations. It was also observed that the gaze of the participants was more fixated on the finishing line. This finding could be explained by the great importance of the positioning of the finishing line in preserving periodontal health, meeting the mechanical and esthetic requirements for fixed prosthetic restorations, and allowing an optimal marginal adaptation [15, 24]. The quality of the finishing line is one of the important criteria used to evaluate a student’s performance in the prosthodontic pre-clinical curriculum [15]. A well-prepared finishing line contributes to the overall retention and resistance of the restoration, which are critical factors in the longevity and success of dental prostheses [15, 23]. These factors may have contributed to the increased fixation on the finishing line observed in the study, as both novices and experts recognize its critical role in ensuring the functional and esthetic success of dental restorations.”

Furthermore, the results showed a significantly longer total duration of fixations (TDF) and time to first fixation (TFF) in the novices compared to the experts. Studies have suggested that a longer total duration of fixation indicates more visual attention and cognitive processing of the stimulus. Thus, the participants tend to spend more time fixating on areas that are more informative or cognitively demanding [22, 25]. Also, the novices displayed a higher number of fixations (NF) compared to the experts possibly indicating that experienced individuals can extract the necessary information with fewer eye movements, reflecting a more focused and efficient search strategy. It has been suggested that experienced individuals rely more on memory to guide attention and can process more of the visual field within each fixation [18].

In the current study, it was observed that both novice and expert participants spent relatively little time examining the adjacent teeth and occlusal reduction when assessing tooth preparations. Instead, their attention was more concentrated on specific Areas of Interest (AOIs) within the tooth preparation itself, rather than on these peripheral aspects. This focus may be attributed to the absence of an opposing tooth, which would typically be used as a reference point for evaluating the necessary clearance to ensure adequate thickness for the prosthetic restoration. Without this reference, participants may have found it challenging to accurately assess the occlusal reduction and the relationship with adjacent teeth, leading them to prioritize other aspects of the preparation [23]. Overall, this gaze behavior may be attributed to the experts’ ability to efficiently identify and focus on the AOIs that are most relevant for assessing tooth preparations, while avoiding unnecessary areas in the displayed images. This selective focus is similar to the gaze behavior observed in novice and expert surgeons, where experienced surgeons can concentrate on the critical site of their surgical activity without being distracted by irrelevant areas. This suggests that with experience, individuals develop a more targeted and efficient visual scanning strategy, allowing them to concentrate on the most important aspects of their task [22].

Previous studies have suggested that a smaller or more stable average pupil diameter can indicate lower cognitive load or stress when individuals perform tasks within their area of expertise [17]. Consequently, studies have shown that experts tend to have a smaller change in pupil size from baseline compared to novices, suggesting a lower cognitive load during task performance. This is because experts have developed more efficient processing strategies and structured schemata in long-term memory, allowing them to handle domain-specific tasks with less mental effort [17]. It was shown that dental radiography experts exhibit smaller pupil size changes when searching for anomalies, indicating a lower cognitive load compared to novices (students) [17]. However, our results do not show any change in the pupil diameter either between the two groups or between the AOIs. It is suggested that both novices and experts may have found the task relatively easy due to their familiarity with it. As a result, the cognitive load required to perform the task might not have been high enough to cause noticeable changes in pupil size [26].

## Conclusion

In conclusion, novices showed longer fixation durations, more fixations, and delayed first fixations on key areas compared to experts, thus supporting the hypothesis. Both groups focused on the buccal wall and margin, although pupil diameter did not differ, suggesting similar cognitive load despite differences in visual engagement.

## Supplementary Information

Supplementary Material 1.

## Data Availability

No datasets were generated or analysed during the current study.
