# Supplementary material for: Eye-tracking metrics to compare visual attention in prosthodontic preclinical evaluations
Source: BMC Oral Health. 2025 Aug 21;25:1350. doi: 10.1186/s12903-025-06708-6 (PMC12369239; doi:10.1186/s12903-025-06708-6)
Supplement: Supplementary file 1 — Supplementary Material 1. [file 12903_2025_6708_MOESM1_ESM.pptx]

## Slide 1
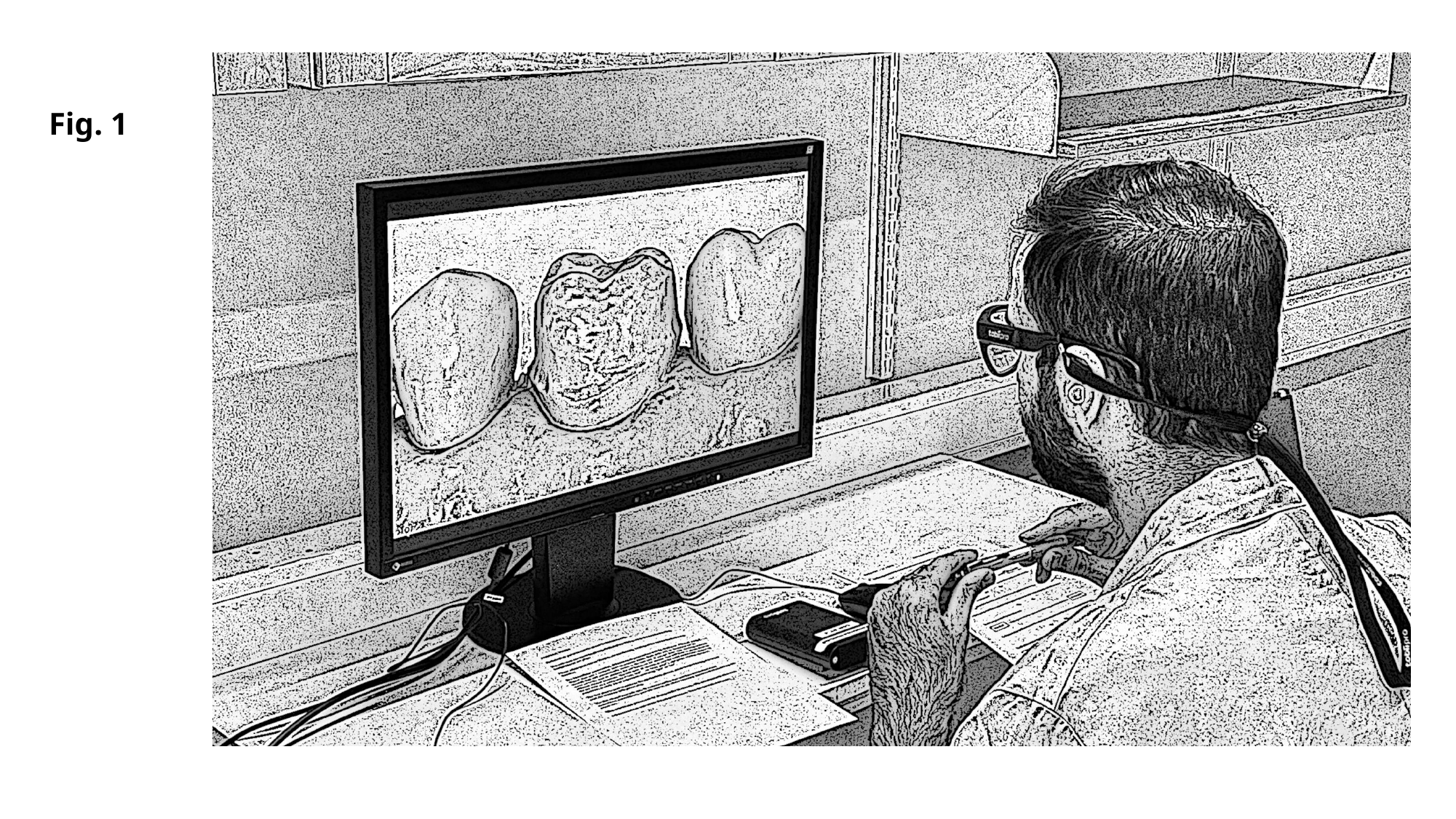

Fig. 1

## Slide 2
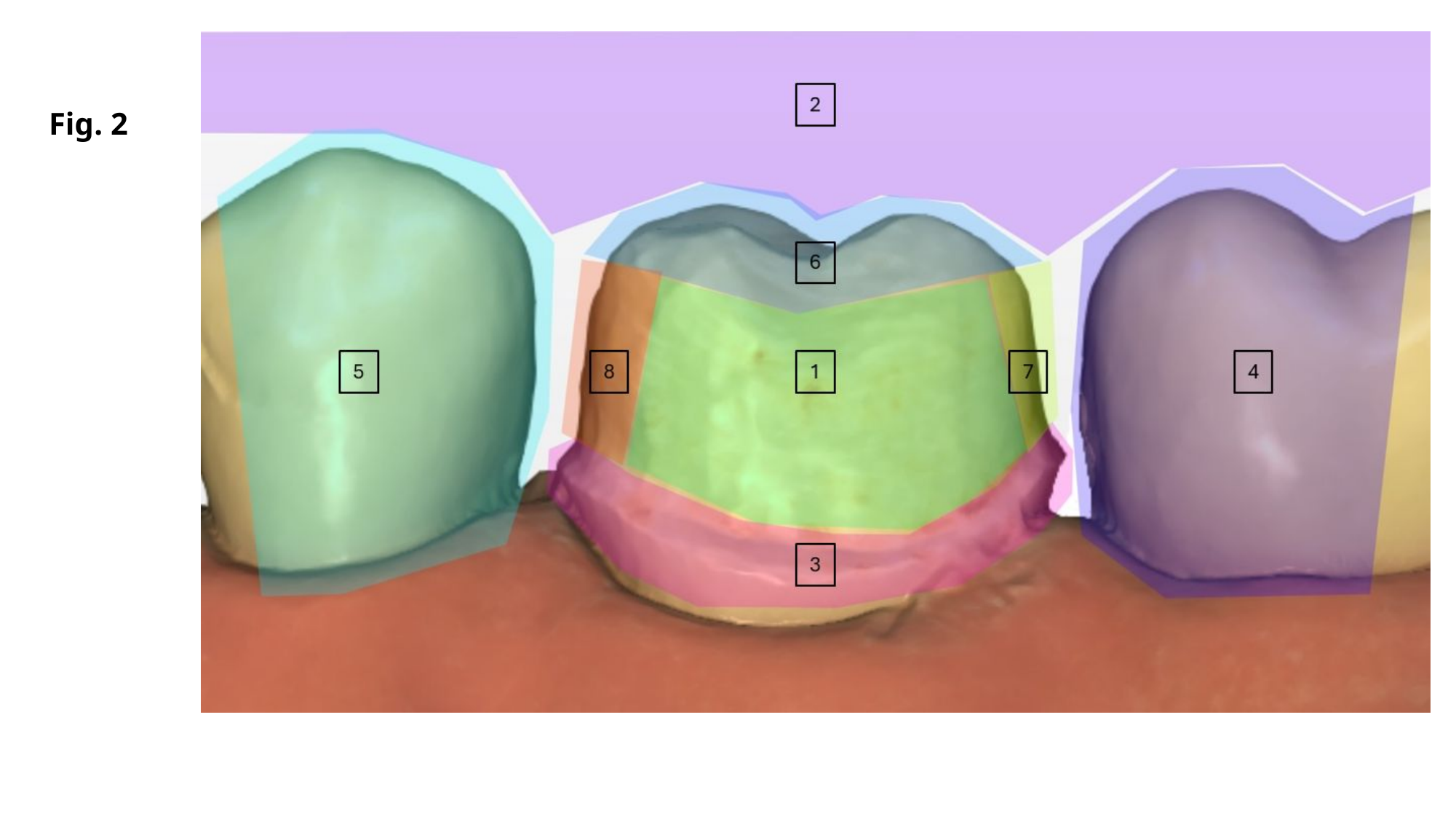

Fig. 2

## Slide 3
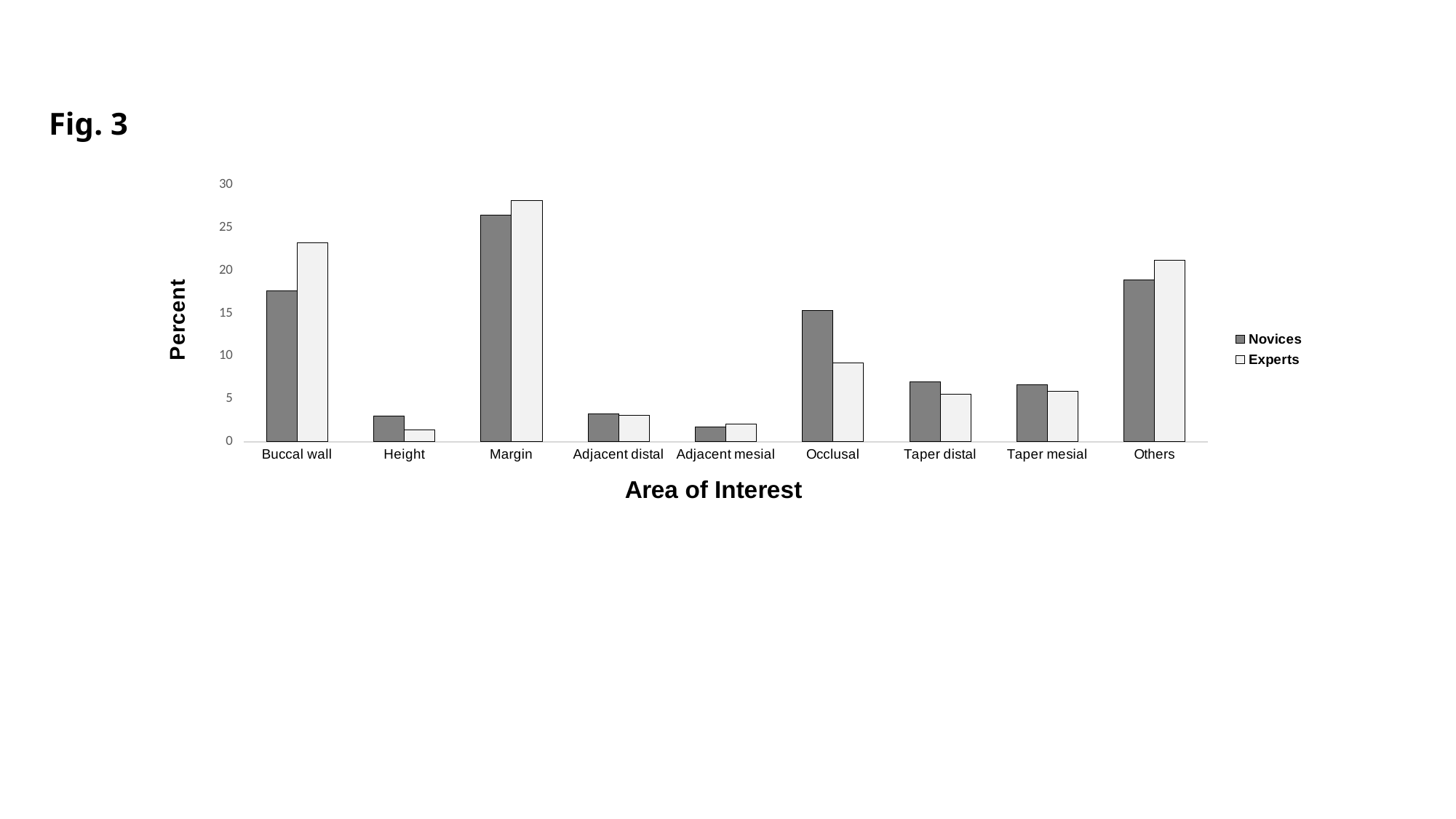

Fig. 3
### Chart
| Category | Novices | Experts |
|---|---|---|
| Buccal wall | 17.6 | 23.2 |
| Height | 3.0 | 1.4 |
| Margin | 26.5 | 28.2 |
| Adjacent distal | 3.3 | 3.1 |
| Adjacent mesial | 1.7 | 2.1 |
| Occlusal | 15.3 | 9.2 |
| Taper distal | 7.0 | 5.6 |
| Taper mesial | 6.7 | 5.9 |
| Others | 18.9 | 21.2 |

## Slide 4
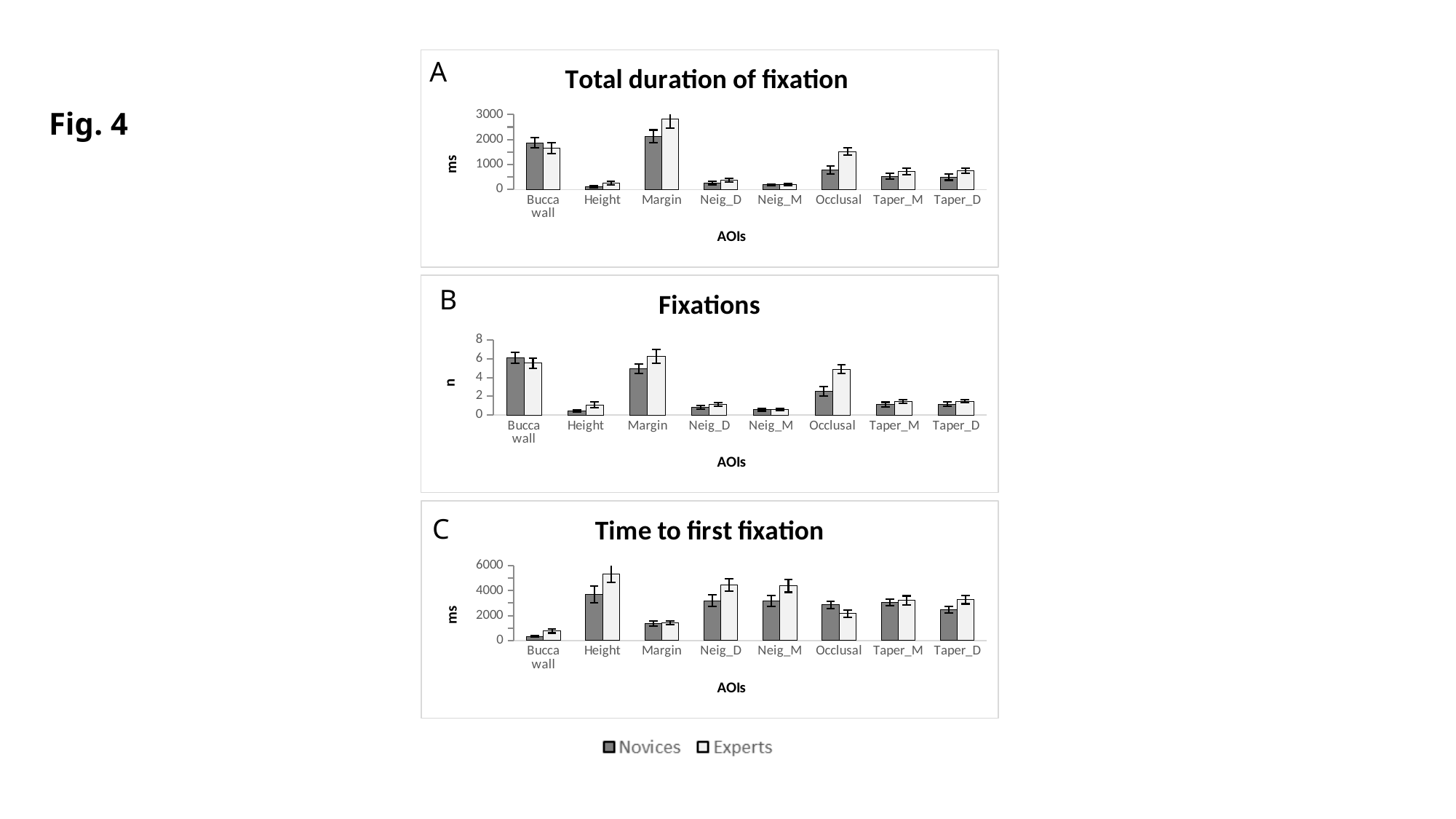

### Chart: Total duration of fixation
| Category | | |
|---|---|---|
| Bucca wall | 1867.1294117647058 | 1654.3805555555557 |
| Height | 115.0764705882353 | 268.40833333333336 |
| Margin | 2125.088235294117 | 2823.933333333334 |
| Neig_D | 268.5441176470588 | 369.4916666666667 |
| Neig_M | 185.61470588235292 | 186.85833333333335 |
| Occlusal | 788.0588235294117 | 1522.0055555555557 |
| Taper_M | 528.0676470588235 | 732.1138888888888 |
| Taper_D | 498.1882352941175 | 754.5861111111111 |A
Fig. 4
### Chart: Fixations
| Category | | |
|---|---|---|
| Bucca wall | 6.105882352941175 | 5.525000000000001 |
| Height | 0.4058823529411764 | 1.061111111111111 |
| Margin | 4.961764705882352 | 6.263888888888889 |
| Neig_D | 0.7852941176470588 | 1.1222222222222225 |
| Neig_M | 0.5470588235294118 | 0.5666666666666668 |
| Occlusal | 2.502941176470588 | 4.883333333333334 |
| Taper_M | 1.1205882352941174 | 1.4083333333333334 |
| Taper_D | 1.1647058823529415 | 1.4472222222222224 |B
D
### Chart: Time to first fixation
| Category | | |
|---|---|---|
| Bucca wall | 337.40091159270725 | 773.8670565302143 |
| Height | 3683.291592920296 | 5338.5493018507295 |
| Margin | 1354.9901877314394 | 1399.5721116359273 |
| Neig_D | 3184.189705882353 | 4455.285958884979 |
| Neig_M | 3185.5058837173024 | 4378.380318755318 |
| Occlusal | 2853.3399537890023 | 2156.7526508264123 |
| Taper_M | 3040.355439787252 | 3215.346961559778 |
| Taper_D | 2462.640931372549 | 3273.732932222051 |C
